# Curcumin Nanoparticles Enhance Mycobacterium bovis BCG Vaccine Efficacy by Modulating Host Immune Responses

**DOI:** 10.1128/IAI.00291-19

**Published:** 2019-10-18

**Authors:** Shaheer Ahmad, Debapriya Bhattacharya, Santosh Kar, Anand Ranganathan, Luc Van Kaer, Gobardhan Das

**Affiliations:** a Special Centre for Molecular Medicine, Jawaharlal Nehru University, New Delhi, India; b Nanoherb Research Laboratory, KIIT TBI, KIIT University, Bhubaneswar, India; c Department of Pathology, Microbiology and Immunology, Vanderbilt University School of Medicine, Nashville, Tennessee, USA; Weill Cornell Medical College

**Keywords:** *Mycobacterium tuberculosis*, BCG vaccine, APCs, memory T cells, K_V_1.3 potassium ion channel, curcumin nanoparticles

## Abstract

Tuberculosis (TB) is one of the deadliest diseases, causing ∼2 million deaths annually worldwide. Mycobacterium bovis bacillus Calmette-Guérin (BCG), the only TB vaccine in common use, is effective against disseminated and meningeal TB in young children but is not effective against adult pulmonary TB. T helper 1 (Th1) cells producing interferon gamma (IFN-γ) and Th17 cells producing interleukin-17 (IL-17) play key roles in host protection against TB, whereas Th2 cells producing IL-4 and regulatory T cells (Tregs) facilitate TB disease progression by inhibiting protective Th1 and Th17 responses. Furthermore, the longevity of vaccine efficacy critically depends on the magnitude of long-lasting central memory T (T_CM_) cell responses. Hence, immunomodulators that promote T_CM_ responses of the Th1 and Th17 cell lineages may improve BCG vaccine efficacy. Here, we show that curcumin nanoparticles enhance various antigen-presenting cell (APC) functions, including autophagy, costimulatory activity, and the production of inflammatory cytokines and other mediators. We further show that curcumin nanoparticles enhance the capacity of BCG to induce T_CM_ cells of the Th1 and Th17 lineages, which augments host protection against TB infection. Thus, curcumin nanoparticles hold promise for enhancing the efficacy of TB vaccines.

## Abstract (precis)

Tuberculosis (TB) is one of the deadliest diseases, causing ∼2 million deaths annually worldwide. Mycobacterium bovis bacillus Calmette-Guérin (BCG), the only TB vaccine in common use, is effective against disseminated and meningeal TB in young children but is not effective against adult pulmonary TB.

## INTRODUCTION

Mycobacterium tuberculosis, the etiological agent of tuberculosis (TB), causes nearly 2 million deaths annually worldwide. One-third of the global population is infected with a latent form of TB, which represents an enormous reservoir waiting for an opportunity to reactivate disease (1). Conditions such as HIV infection that impair immunity may lead to such TB reactivation (2). In the absence of HIV coinfection, only 5 to 10% of latently infected individuals develop TB in their lifetime, whereas 30% of coinfected individuals develop active TB (3). Consequently, a substantial number of deaths in HIV patients are associated with TB infection (4–6). Although one-third of the global population is latently infected with M. tuberculosis, the vast majority of individuals are resistant to TB, despite repeated exposure. It is well established that host immune responses play a central role in host resistance against TB. CD4^+^ T helper (Th) cells play a key role in protective immunity against TB (7). Previous reports, including studies by our group, have demonstrated that Th1 cells play an important role in host resistance against TB infection (8, 9). However, while the vast majority of TB patients mount Th1 responses, disease continues to progress in many patients (10). These observations indicate that Th1 cell responses are essential but not sufficient for disease protection.

Mycobacterium bovis bacillus Calmette-Guérin (BCG) is the only TB vaccine mainly effective against disseminated and meningeal TB in young children; it is likely that the host-protective immune responses that it induces diminish over time (11–14). It is well established that long-term memory responses are critically dependent on central memory T (T_CM_) cells rather than effector memory T (T_EM_) cells (15). BCG induces both T_CM_ and T_EM_ responses, where T_EM_ responses may play a role in eliminating virulent strains and T_CM_ responses protect against childhood TB but diminish over time. It is well known that BCG immunization promotes the generation of T_EM_ cells, which are the relevant source of T effector cells. These cells predominantly reside in peripheral organs (i.e., the site of infection). However, when the antigen load is gradually reduced, these cells undergo apoptosis. In contrast, T_CM_ cells mostly reside within lymphoid organs and represent a pool of memory T cells for future antigen challenge. Upon antigen reexposure, these cells rapidly proliferate and convert into T_EM_ cells. Heightened production of T_EM_ and T effector cells correlates with the presence of high antigen loads (15). In areas where TB is endemic, the level of exposure to various environmental mycobacteria is also very high. Due to this continuous exposure, most T_CM_ cells differentiate into T_EM_ and T effector cells. Moreover, continuous exposure to environmental antigens eventually causes anergy and exhaustion of antigen-specific T cells (16–18). Consistent with these findings, it is perhaps not surprising that BCG protects children against disseminated TB yet provides little protection against pulmonary TB in adults, who are continuously exposed to environmental mycobacteria (19). Therefore, enhancing the magnitude of T_CM_ cell responses holds promise for improving long-lasting vaccine efficacy. T_CM_ and T_EM_ cells are in a dynamic balance, and their ratio impacts vaccine efficacy. Hence, altering the ratio of these two memory T cell subsets provides the opportunity to improve BCG vaccine efficacy. Recently, we reported that simultaneous inhibition of regulatory T cell (Treg) and Th2 cell responses by immunomodulators in BCG-vaccinated mice promotes T_CM_ cells of the Th1 lineage that protect against M. tuberculosis infection (20). We subsequently showed that inhibition of the potassium channel K_V_1.3, which is predominantly expressed by T_EM_ cells, by clofazimine enhances T_CM_ responses in BCG-immunized mice and provides long-term protection against TB infection (21). According to WHO guidelines, clofazimine can be used for the treatment of multiple-drug-resistant (MDR) TB. However, clofazimine has serious limitations, which include immune suppression and long-lasting accumulation in various organs (22, 23). Curcumin is known for its immunomodulatory properties and exhibits efficacy against several diseases. However, due to its poor intestinal absorption, rapid metabolism, and rapid systemic elimination, bioavailability is poor, limiting its clinical use. To overcome this limitation, we have generated a nanoparticle-formulated version of curcumin (24). Previously, we reported that our formulation of curcumin nanoparticles possesses five-times-higher bioavailability in mice (24). Here, we explored the immunomodulatory properties of curcumin nanoparticles during BCG immunization and observed a dramatic improvement in the T_CM_/T_EM_ cell ratios of host-protective Th1 and Th17 cell responses. These properties of curcumin nanoparticles were associated with the upregulation of effector functions in macrophages and dendritic cells. Moreover, these activated antigen-presenting cells (APCs) produced copious amounts of interleukin-12 (IL-12) and NO that promoted bacterial clearance and expansion of memory T cells.

## RESULTS

### Curcumin nanoparticles enhance the efficacy of BCG immunization in a murine TB model.

**FIG 1:**
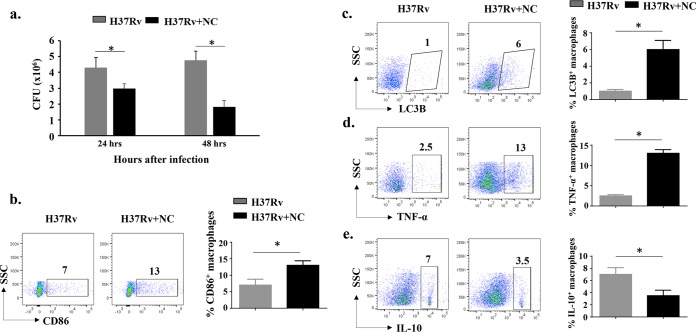
*In vitro* effects of nanocurcumin on macrophages. Six- to eight-week-old C57BL/6 mice were given an i.p. injection of 2 ml thioglycolate medium (4%). After 5 days, macrophages were obtained by peritoneal lavage. Macrophages were washed once with cold PBS and suspended in cold RPMI 1640 medium. Cells were counted, seeded on 12-well plates, and maintained at 37°C in RPMI 1640 medium supplemented with penicillin-streptomycin (1,000 U/ml) and 10% heat-inactivated fetal calf serum. Cells were washed with culture medium every 6 h for a period of 24 h. After overnight incubation, nonadherent cells were washed. Adherent cells were infected with H37Rv at a ratio of 10:1. Cells were then treated with 60 nM nanocurcumin (NC) and kept at 37°C in a CO_2_ incubator. After 24 and 48 h of infection, macrophages were harvested for CFU determination and flow cytometry. (a) CFU counts from peritoneal macrophages at 24 and 48 h. (b) Activation status of macrophages, as shown by pseudocolor plots and bar diagrams of CD11b^+^ MHCII^+^ CD86^+^ cells. (c) Autophagy status of macrophages as shown by pseudocolor plots and bar diagrams of CD11b^+^ MHCII^+^ LC3B^+^ cells. (d) TNF-α-producing status of macrophages, as shown by pseudocolor plots and bar diagrams of TNF-α-secreting CD11b^+^ MHCII^+^ macrophages. (e) IL-10-producing status of macrophages, as shown by pseudocolor plots and bar diagrams of IL-10-secreting CD11b^+^ MHCII^+^ macrophages. All data are representative of results from 3 independent experiments. All values are represented as means ± SD. Statistical analyses were done by ANOVA with Tukey’s *post hoc* analysis. * denotes a *P* value of ≤0.05. Experimental groups are (i) H37Rv and (ii) H37Rv plus nanocurcumin. SSC, side scatter.

BCG is the only vaccine commonly used for TB but has limited efficacy in adults. However, it is sufficiently effective against disseminated and meningeal TB in young children (9, 11–13). Therefore, it is likely that host-protective immune responses diminish over time. To explore the immunomodulatory properties of curcumin nanoparticles (nanocurcumin), we performed a series of *in vitro* experiments with peritoneal macrophages (see Materials and Methods for details). We tested the capacity of nanocurcumin to modulate BCG vaccine efficacy. First, we tested the effects of nanocurcumin on M. tuberculosis H37Rv-infected macrophages. We observed that nanocurcumin treatment of H37Rv-infected peritoneal macrophages reduces the bacterial burden in a time-dependent fashion (Fig. 1a). Autophagy is the prime mechanism employed by macrophages to eliminate M. tuberculosis infection (25). LC3b is a marker that identifies the status of autophagy in cells (25). Therefore, we profiled the percentage of autophagy as well as the activation of macrophages, which revealed that nanocurcumin treatment significantly enhances cellular activation and autophagy (Fig. 1b and c). IL-10 polarizes macrophages toward immunosuppressive phenotypes, whereas tumor necrosis factor alpha (TNF-α) contributes to the activation of macrophages (26, 27). Interferon gamma (IFN-γ) and TNF-α act in conjunction on infected macrophages to induce the production of NO and other free radicals to clear bacteria. IL-10 production downregulates the expression of major histocompatibility complex (MHC) class II (MHCII) and costimulatory molecules, which suppresses protective immune responses. We found that nanocurcumin treatment significantly upregulated the levels of TNF-α and downregulated the levels of IL-10 in H37Rv-infected macrophages (Fig. 1d and e).

**FIG 2:**
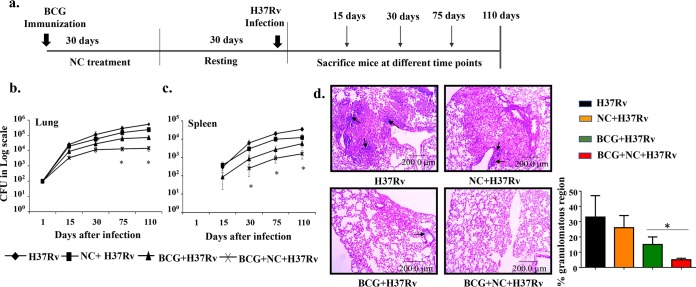
Nanocurcumin enhances BCG vaccine efficacy in H37Rv-challenged mice. Mice were distributed into 4 groups: (i) naive C57BL/6 mice, (ii) nanocurcumin (NC)-treated mice, (iii) BCG-immunized and PBS-treated mice, and (iv) BCG-immunized and nanocurcumin-treated mice. (a) Schematic representation of the experiment. Mice were immunized with BCG (subcutaneously) and then injected with nanocurcumin for 30 days, followed by a resting period of another 30 days. Mice were then challenged with H37Rv via the aerosol route, with a low-dose inoculum of approximately 110 CFU per mouse. After that, mice were euthanized at various time points (15, 30, 75, and 110 days), and lungs and spleen were harvested and assessed for bacterial burden. (b) CFU counts in lungs at different time intervals. (c) CFU counts in spleen at different time intervals. (d, left) Photomicrographs of lung histological sections (6 μm) of different experimental groups after 75 days of infection, stained with hematoxylin and eosin. Arrows denote the granulomatous region. (Right) Graphical representation of the percentages of granulomatous regions in different experimental groups. All data are representative of results from 3 independent experiments with 5 mice from each experimental group at each time point. All values are represented as means ± SD. Statistical analyses were done by ANOVA with Tukey’s *post hoc* analysis. In panels b and c, comparisons were done between the group receiving BCG, nanocurcumin, and H37Rv and all other experimental groups. * denotes a *P* value of ≤0.05. Experimental groups are (i) H37Rv, (ii) nanocurcumin plus H37Rv, (iii) BCG plus H37Rv, and (iv) BCG plus nanocurcumin and H37Rv.

After establishing its immunomodulatory properties *in vitro*, we tested the capacity of nanocurcumin to modulate BCG vaccine efficacy in a murine model of TB. We immunized C57BL/6 mice with BCG subcutaneously and subsequently treated the animals with nanocurcumin for 30 days, followed by a resting period of 30 days. These animals were then aerosol challenged with a virulent strain of H37Rv at a low dose (approximately 110 CFU) (Fig. 2a). The bacterial burdens in lungs and spleen were measured at various time points. We observed that nanocurcumin treatment enhanced BCG vaccine efficacy, as determined by the significant reduction of bacterial loads in both lungs and spleen (Fig. 2b and c). Furthermore, histological studies revealed that nanocurcumin treatment reduced the granulomatous regions in BCG-immunized mice (Fig. 2d).

### Nanocurcumin treatment enhances immunity against BCG by modulating innate immune responses.

**FIG 3:**
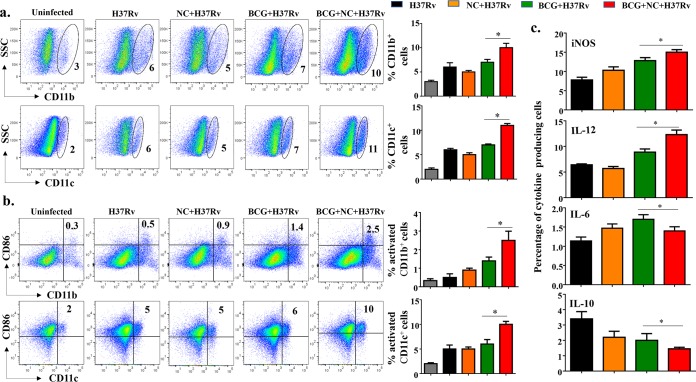
Nanocurcumin activates APCs in lungs of BCG-vaccinated mice. Mice were treated as described in the legend to Fig. 2a. At 75 days postinfection, lungs were harvested, and single-cell suspensions were made and cultured overnight, followed by staining and flow cytometry. Primarily monocytes were gated based on forward scatter versus side scatter (SSC), and monocytes and other cell types were identified by specific antibodies. (a) Pseudocolor plot and bar diagrams of CD11b^+^ and CD11c^+^ APCs in lungs. (b) Activation status of APCs. Pseudocolor plots and bar diagrams of CD11b^+^ CD86^+^ and CD11c^+^ CD86^+^ APCs in lungs are shown. (c) Bar diagrams of IL-12-, IL-10-, IL-6-, and iNOS-producing APCs. All data are representative of results from 3 independent experiments with 5 mice from each experimental group at each time point. All values are represented as means ± SD. Statistical analyses were done by ANOVA with Tukey’s *post hoc* analysis. * denotes a *P* value of ≤0.05. Experimental groups are (i) uninfected, (ii) H37Rv, (iii) nanocurcumin (NC) plus H37Rv, (iv) BCG plus H37Rv, and (v) BCG plus nanocurcumin and H37Rv.

M. tuberculosis successfully survives and replicates within phagocytic cells by altering host immune responses (26, 28). M. tuberculosis has developed a variety of strategies for this purpose, which include blockade of phagosome maturation and phagolysosome fusion and inhibition of autophagy. It is well known that nanocurcumin induces autophagy in human macrophages and helps in antigen priming of T cells (29–31). M. tuberculosis modulates antigen presentation and the expression of costimulatory molecules, which diminishes host-protective immune responses. In accordance with these findings, we found that at 75 days postinfection, nanocurcumin treatment upregulates the function of CD11b^+^ and CD11c^+^ APCs that facilitate T cell priming. We found that the numbers of CD11b^+^ and CD11c^+^ cells were significantly increased in the lungs of animals treated with nanocurcumin during BCG immunization (Fig. 3a). We further found that nanocurcumin treatment enhances the expression of CD86 in both CD11b^+^ and CD11c^+^ cells in lungs of BCG-immunized mice (Fig. 3b), suggesting that nanocurcumin creates an environment conducive for priming and activating T cells. Next, we examined cytokines and effector molecules in macrophages from these animals. We found that nanocurcumin upregulated the expression of inducible nitric oxide synthase (iNOS) and IL-12 (Fig. 3c). In contrast, levels of IL-6 and IL-10 were reduced (Fig. 3c). Therefore, these results indicated that nanocurcumin induces an environment that promotes priming and activation of host-protective T cells during BCG immunization.

### Nanocurcumin treatment increases the proliferation and activation of antigen-specific CD4^+^ and CD8^+^ T cells in BCG-immunized animals.

**FIG 4:**
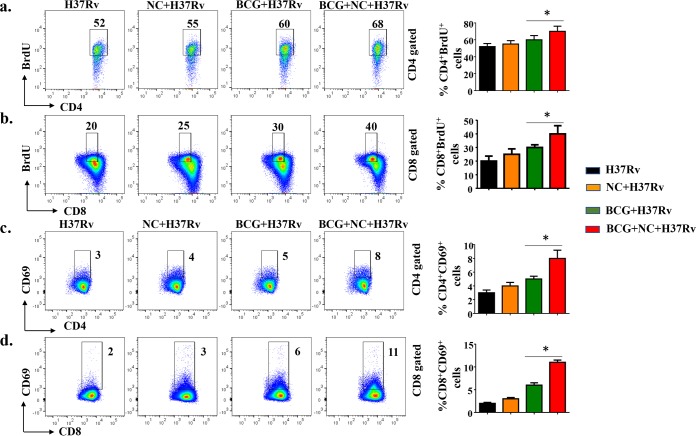
Nanocurcumin treatment increases the proliferation and activation of antigen-specific CD4^+^ and CD8^+^ T cells in BCG-immunized animals. Mice were treated as described in the legend to Fig. 2a. At 72 days postinfection, mice were injected with BrdU. Seventy-two hours after injection of BrdU, spleens were harvested, and single-cell suspensions were made. Cells were cultured overnight with M. tuberculosis lysate (CSA) stimulation to assess antigen-specific immune responses. These cells were stained with anti-CD3, -CD4, -CD8, -CD69, and -BrdU antibodies, followed by flow cytometry. For FACS analysis, we first isolated the CD3 and then the CD4 or CD8 populations, and within those populations, we then gated BrdU^+^ and CD69^+^ populations. (a) CD4 T cell proliferative status, as shown by pseudocolor and bar diagrams of CD4^+^ BrdU^+^ T cells. (b) CD8 T cell proliferative status, as shown by pseudocolor plots and bar diagrams of CD8^+^ BrdU^+^ T cells. (c) CD4 T cell activation status, as shown by pseudocolor plots and bar diagrams of CD4^+^ CD69^+^ T cells. (d) CD8 T cell activation status, as shown by pseudocolor plots and bar diagrams of CD8^+^ CD69^+^ T cells. All data are representative of results from 3 independent experiments with 5 mice from each experimental group at each time point. All values are represented as means ± SD. Statistical analyses were done by ANOVA with Tukey’s *post hoc* analysis. * denotes a *P* value of ≤0.05. Experimental groups are (i) H37Rv, (ii) nanocurcumin (NC) plus H37Rv, (iii) BCG plus H37Rv, and (iv) BCG plus nanocurcumin and H37Rv.

The activation of CD11b^+^ and CD11c^+^ cells in nanocurcumin-treated animals prompted us to examine possible alterations in T lymphocyte responses. It is well established that both CD4^+^ and CD8^+^ T cells play important roles in host resistance against TB (32). Antigen-specific CD4^+^ T cells producing IFN-γ and TNF-α act on infected macrophages to promote bacterial clearance via autophagy (25). To determine whether nanocurcumin treatment increases the activation and proliferation of T cells in BCG-immunized mice, we injected animals with bromodeoxyuridine (BrdU) prior to sacrifice at 75 days postinfection. Splenocytes were harvested and challenged with M. tuberculosis-derived complete soluble antigen (CSA) to estimate antigen-specific activation and proliferative responses. Our results demonstrated that splenocytes from BCG-immunized mice that received nanocurcumin treatment exhibited increased proliferative responses for both CD4^+^ and CD8^+^ T cells compared with the other experimental groups of mice (Fig. 4a and b). Consistent with these findings, both CD4^+^ and CD8^+^ T cells upregulated the early activation marker CD69 in nanocurcumin- and BCG-treated mice (Fig. 4c and d). These results suggested that nanocurcumin treatment increased the proliferation and activation of both CD4^+^ and CD8^+^ T cells in BCG-immunized mice.

### Nanocurcumin improves the T_CM_-to-T_EM_ cell ratio in BCG-immunized mice.

**FIG 5:**
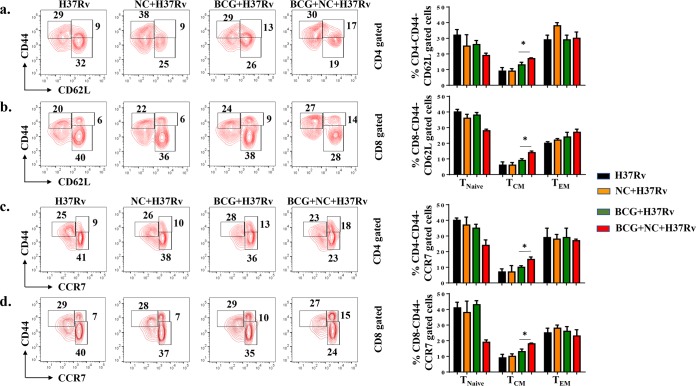
Nanocurcumin enhances BCG vaccine efficacy by increasing the T_CM_/T_EM_ cell ratio in spleen. Mice were treated as described in the legend to Fig. 2a. At 75 days postinfection, spleens were harvested, and single-cell suspensions were made. Cells were cultured overnight with the M. tuberculosis lysate (CSA) to assess antigen-specific immune responses. These cells were stained with anti-CD3, -CD4, -CD8, -CD44, -CD62L, and -CCR7 antibodies, followed by flow cytometry. Different memory subsets, including naive T cells (CD4^+^ CD44^−^ CD62L^−^/CD4^+^ CD44^−^ CCR7^−^ or CD8^+^ CD44^−^ CD62L^−^/CD8^+^ CD44^−^ CCR7^−^), T_CM_ cells (CD4^+^ CD44^+^ CD62L^+^/CD4^+^ CD44^+^ CCR7^+^ or CD8^+^ CD44^+^ CD62L^+^/CD8^+^ CD44^+^ CCR7^+^), and T_EM_ cells (CD4^+^ CD44^+^ CD62L^−^/CD4^+^ CD44^+^ CCR7^−^ or CD8^+^ CD44^+^ CD62L^−^/CD8^+^ CD44^+^ CCR7^−^), were analyzed by flow cytometry. (a) Contour plots and bar diagrams of different (CD62L-gated) CD4^+^ T memory subsets in spleens of different experimental groups. (b) Contour plots and bar diagrams of different (CD62L-gated) CD8^+^ T memory subsets in spleens of different experimental groups. (c) Contour plots and bar diagrams of different (CCR7-gated) CD4^+^ T memory subsets in spleens of different experimental groups. (d) Contour plots and bar diagrams of different (CCR7-gated) CD8^+^ T memory subsets in spleens of different experimental groups. All data are representative of results from 3 independent experiments with 5 mice from each experimental group at each time point. All values are represented as means ± SD. Statistical analyses were done by ANOVA with Tukey’s *post hoc* analysis. * denotes a *P* value of ≤0.05. Experimental groups are (i) H37Rv, (ii) nanocurcumin (NC) plus H37Rv, (iii) BCG plus H37Rv, and (iv) BCG plus nanocurcumin and H37Rv.

**FIG 6:**
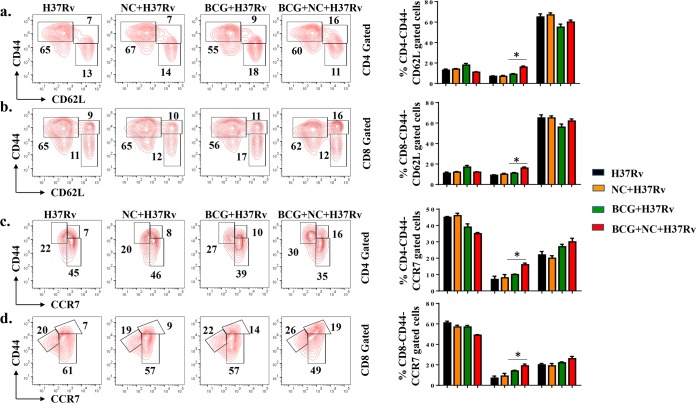
Nanocurcumin enhances the T_CM_/T_EM_ cell ratio in lungs of BCG-immunized mice. Mice were treated as described in the legend to Fig. 2a. At 75 days postinfection, lungs were harvested, and single-cell suspensions were made. Cells were cultured overnight with the M. tuberculosis lysate (CSA) and stained to assess antigen-specific immune responses. Cells were stained with anti-CD3, -CD4, -CD8, -CD44, -CD62L, and -CCR7 antibodies, followed by flow cytometry. Different memory subsets, including naive T cells (CD4^+^ CD44^−^ CD62L^−^/CD4^+^ CD44^−^ CCR7^−^ or CD8^+^ CD44^−^ CD62L^−^/CD8^+^ CD44^−^ CCR7^−^), T_CM_ cells (CD4^+^ CD44^+^ CD62L^+^/CD4^+^ CD44^+^ CCR7^+^ or CD8^+^ CD44^+^ CD62L^+^/CD8^+^ CD44^+^ CCR7^+^), and T_EM_ cells (CD4^+^ CD44^+^ CD62L^−^/CD4^+^ CD44^+^ CCR7^−^ or CD8^+^ CD44^+^ CD62L^−^/CD8^+^ CD44^+^ CCR7^−^), were analyzed by flow cytometry. (a) Contour plots and bar diagrams of different (CD62L-gated) CD4^+^ T memory subsets in lungs of different experimental groups. (b) Contour plots and bar diagrams of different (CD62L-gated) CD8^+^ T memory subsets in lungs of different experimental groups. (c) Contour plots and bar diagrams of different (CCR7-gated) CD4^+^ T memory subsets in spleens of different experimental groups. (d) Contour plots and bar diagrams of different (CCR7-gated) CD8^+^ T memory subsets in spleens of different experimental groups. All data are representative of results from 3 independent experiments with 5 mice from each experimental group at each time point. All values are represented as means ± SD. Statistical analyses were done by ANOVA with Tukey’s *post hoc* analysis. * denotes a *P* value of ≤0.05. Experimental groups are (i) H37Rv, (ii) nanocurcumin (NC) plus H37Rv, (iii) BCG plus H37Rv, and (iv) BCG plus nanocurcumin and H37Rv.

Long-term vaccine efficacy primarily depends upon the ratio of T_CM_ to T_EM_ cells generated during infection (15, 19). Previous studies have shown that BCG does not induce sufficient levels of T_CM_ cells in lungs and spleen (16–18). Therefore, a strategy that enhances T_CM_ responses may provide improved vaccine efficacy. K_V_1.3 is a potassium channel predominantly expressed on T_EM_ cells and is required for the differentiation of T_CM_ cells into T_EM_ cells (33, 34). Hence, blocking the K_V_1.3 channel may amplify CD4^+^ and CD8^+^ T_CM_ cells. In fact, such a blockade has recently been shown to increase T_CM_ cells (21, 35). Nanocurcumin blocks the K_V_1.3 channel and is effectively distributed to lungs and spleen. Therefore, to elucidate the effect of K_V_1.3 blockade by nanocurcumin on BCG immunization, we analyzed the phenotype of T_CM_ and T_EM_ cells by flow cytometry 75 days after aerosol challenge. We found that nanocurcumin treatment in BCG-immunized mice yielded higher numbers of T_CM_ (CD44^hi^ CD62L^hi^ and CD44^hi^ CCR7^hi^) cells than T_EM_ (CD44^hi^ CD62L^low^ and CD44^hi^ CCR7^low^) cells in the CD4^+^ T cell subset (Fig. 5a and c) than in BCG-immunized and unimmunized mice. A similar trend in the prevalence of T_CM_ (CD44^hi^ CD62L^hi^ and CD44^hi^ CCR7^hi^) and T_EM_ (CD8^+^ CD44^hi^ CD62L^low^ and CD44^hi^ CCR7^low^) populations was found for CD8^+^ T cells (Fig. 5b and d). Interestingly, the magnitude of the increase in CD8^+^ T_CM_ cells was more pronounced than for CD4^+^ T_CM_ cells. Next, we examined the status of memory T cells in lungs and found a trend similar to that in the spleen (Fig. 6). Therefore, nanocurcumin treatment modulates T lymphocyte memory responses, especially the T_CM_ cell pool. This enhanced T_CM_/T_EM_ cell ratio induced by nanocurcumin treatment might provide long-term protection from TB (see Table S1 in the supplemental material).

### Nanocurcumin promotes antigen-specific, host-protective immune responses in BCG-immunized mice.

**FIG 7:**
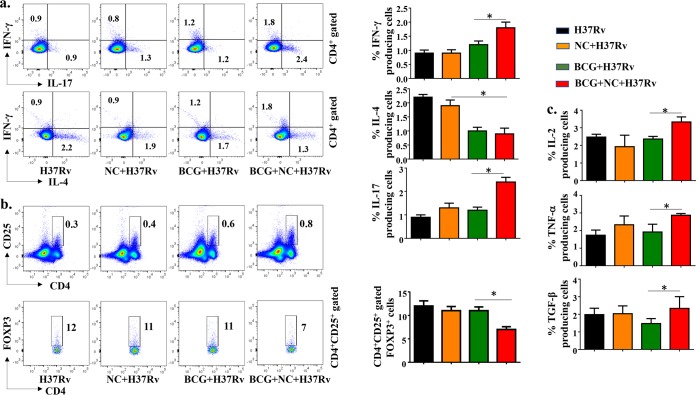
Nanocurcumin treatment in BCG-immunized mice enhances protective Th1 and Th17 cells and inhibits Th2 cells and Tregs. Mice were treated as described in the legend to Fig. 2a. At 75 days postinfection, spleens were harvested, and single-cell suspensions were made. Cells were cultured overnight with the M. tuberculosis lysate (CSA); stained for anti-CD3, -CD4, -IFN-γ, -IL-4, -IL-17, -CD25, -FoxP3, -TNF-α, -IL-2, and -TGF-β antibodies; and analyzed by flow cytometry. (a) Pseudocolor plots and bar diagrams for IFN-γ-, IL-4-, and IL-17-producing CD4 T cells. (b) Pseudocolor plots and bar diagrams for CD4^+^ CD25^+^ FoxP3^+^ T cells in different experimental groups. (c) Bar diagrams of TNF-α, IL-2, and TGF-β cytokine-producing cells in splenocytes of different experimental groups. All data are representative of results from 3 independent experiments with 5 mice from each experimental group at each time point. All values are represented as means ± SD. Statistical analyses were done by ANOVA with Tukey’s *post hoc* analysis. * denotes a *P* value of ≤0.05. Experimental groups are (i) H37Rv, (ii) nanocurcumin (NC) plus H37Rv, (iii) BCG plus H37Rv, and (iv) BCG plus nanocurcumin and H37Rv.

Our finding that nanocurcumin enhances the T_CM_/T_EM_ cell ratio prompted us to assess the cytokine production profile of T cells. It is well established that Th1 responses are indispensable for host protection against TB, and emerging evidence indicates an important role for Th17 cells as well (36). As Th cell subsets are in a dynamic balance, Th2 and Treg responses are enhanced after M. tuberculosis infection, which inhibits host-protective Th1 and Th17 responses. Hence, long-term protection is possible only by blocking these unfavorable T cell responses. Recently, we have shown that simultaneous inhibition of Th2 cells and Tregs not only favors Th1 responses but also promotes T_CM_ responses (20). Therefore, enhancing BCG vaccine efficacy may be achieved by enhancing both Th1 and Th17 responses and simultaneously suppressing Th2 and Treg responses. Nanocurcumin satisfies these criteria and, hence, could be useful in enhancing BCG vaccine efficacy. To elucidate the effect of nanocurcumin treatment on immune responses elicited following BCG immunization, splenocytes were stimulated with M. tuberculosis-derived CSA, and the frequencies of CD4^+^ T cells producing the signature cytokines associated with Th1 (IFN-γ), Th2 (IL-4), and Th17 (IL-17) cells were determined. Our observations demonstrated that nanocurcumin treatment in BCG-immunized mice significantly increased IFN-γ-producing Th1 and IL-17-producing Th17 cells, whereas the levels of IL-4-producing Th2 cells were moderately decreased (Fig. 7a). Because CD4^+^ CD25^+^ FoxP3^+^ Tregs favor disease pathogenesis, we also measured the status of these cells (CD4^+^ CD25^+^ Foxp3^+^). Remarkably, we found a significant decrease of Tregs in nanocurcumin-treated, BCG-immunized mice compared with BCG-immunized and untreated animals (Fig. 7b).

To identify the innate cytokines that are responsible for this biased T cell polarization, we determined the levels of IL-2-, TNF-α-, and transforming growth factor β (TGF-β)-producing cells (Fig. 7c and Fig. S1). IL-2, IFN-γ, and TNF-α are hallmarks of polyfunctional memory cells (37). We found that levels of TNF-α- and IL-2-producing cells were increased, with a concomitant increase in the level of IFN-γ-producing cells (Fig. 7a and c). These results indicated that nanocurcumin treatment enhances Th1 and Th17 cell responses but restricts Th2 cell and Treg responses in BCG-immunized mice.

## DISCUSSION

M. tuberculosis evades host immune responses in part by altering the balance of T cell responses. BCG is the only TB vaccine available, but its efficacy in preventing adult pulmonary TB is limited, varying from 0 to 80%, depending on the ethnicity and geographical location of the vaccinated population (11–14). Although the precise immune responses required for optimal host protection are still unclear, it is well established that Th1 and Th17 responses play key roles, whereas Th2 cells and Tregs promote disease progression by inhibiting host-protective Th1 and Th17 responses (20, 36). BCG induces adequate levels of IFN-γ-producing T cells in the host but generates only a few IL-17-producing T cells. Moreover, recent studies have shown that CD4^+^ cell-derived IFN-γ is not the limiting factor, as excessive levels of IFN-γ may have deleterious inflammatory effects (38). Concurrently, BCG induces TGF-β and IL-10 production, which facilitates Treg and Th2 cell differentiation (39–41). Additionally, mesenchymal stem cells (MSCs) also induce Tregs and suppress protective immune responses (42, 43). APCs such as macrophages and dendritic cells engulf the bacteria at the site of infection and subsequently migrate to secondary lymphoid organs. These cells also upregulate costimulatory molecules, produce proinflammatory cytokines, and present antigens to T cells, which then differentiate into distinct effector cell subsets (26). BCG immunization induces IL-10 production, which downregulates the expression of MHC class II and costimulatory molecules (44–47), limiting the efficacy of the BCG vaccine. It was previously reported that inhibition of IL-10 increases the efficacy of BCG (48). Our results demonstrate that the addition of nanocurcumin to the BCG vaccine inhibits the production of immunosuppressive cytokines, such as IL-10, in infected macrophages.

Vaccine efficacy is critically dependent on T helper memory subsets. T_EM_ cells have a limited capacity to proliferate and differentiate but quickly respond to antigenic challenge by converting into terminal effector cells. On the other hand, T_CM_ cells are capable of extensive proliferation, are comparatively less differentiated than T_EM_ cells, and persist for only a short time period. In response to antigenic challenge, many T_CM_ cells differentiate into effector cells, some differentiate into T_EM_ cells (15), and some cells retain their T_CM_ phenotype for future immune responses. T_CM_ cells have the ability to respond to two types of signals: (i) homeostatic signals for self-renewal and (ii) antigen-specific signals for differentiation into T_EM_ cells. BCG induces antigen-specific T_EM_ cells and limited levels of T_CM_ cells (19). Moreover, although BCG induces sufficient numbers of T_CM_ cells in the lungs to permit the generation of T_EM_ cells, these cells become gradually exhausted due to continuous exposure to environmental mycobacteria found in countries with a high TB burden (21). Therefore, the ratio of T_CM_ to T_EM_ cells is critically important for BCG vaccine efficacy. Our study revealed that nanocurcumin increases the T_CM_ pool without substantially altering the T_EM_ pool, which significantly improved the ratio of T_CM_ over T_EM_ responses in BCG-immunized animals.

Our results implied that nanocurcumin treatment effectively activated APCs by upregulating costimulatory molecules, induced IL-12 and iNOS production, and reduced the production of IL-10 in APCs. We propose that these alterations induced by nanocurcumin established a physiological environment conducive for the generation of protective T cell responses against TB (26, 49–52).

In addition to its effects on APCs, *in vivo* T cell activation and proliferation studies with BrdU clearly demonstrated that nanocurcumin enhanced the proliferation of CD4 and CD8 T cells in BCG-immunized animals. In this context, it was previously established that nanocurcumin prevents AICD (activation-induced cell death) of antigen-specific T cells induced during directly observed treatment, short term (DOTS) (53). Therefore, nanocurcumin enhances adaptive immune responses by inducing M. tuberculosis-specific activated T cells.

Curcumin blocks the K_V_1.3 potassium channel (54), which is predominantly expressed on T_EM_ cells (35, 55). Although T_EM_ cells play a role in bacterial clearance, they are also the major cause of tissue-specific inflammation (56). A number of studies have shown that K_V_1.3 blockade can inhibit inflammation in a variety of diseases (23). Moreover, K_V_1.3 loss of function in mice causes a delay in T_CM_-to-T_EM_ cell conversion and promotes the generation of a pool of long-lasting T_CM_ cells (57). Consistent with these findings, we found that nanocurcumin increased the pool of CD4^+^ and CD8^+^ T_CM_ cells in spleen and lungs. No significant changes were observed in T_EM_ cells, indicating that nanocurcumin inhibits the conversion of T_CM_ to T_EM_ cells and increases the T_CM_/T_EM_ cell ratio, thereby enhancing BCG vaccine efficacy and reducing disease pathology.

### Conclusion.

Altogether, we have shown that nanocurcumin activates the innate part of the immune response to induce costimulatory molecules and proinflammatory cytokines in APCs. Simultaneously, it also increases autophagy in APCs. These findings are consistent with the enhanced antigen-presenting capacity of nanocurcumin-treated APCs (29). In the adaptive part of the immune response, nanocurcumin restricts the conversion of T_CM_ to T_EM_ cells to increase the T_CM_ pool for the establishment of long-term memory responses to the BCG vaccine. Additionally, nanocurcumin protects tissues against uncontrolled inflammation. The precise molecular mechanism by which nanocurcumin enhances APC and T cell functions will require further studies.

## MATERIALS AND METHODS

### Ethics statement.

All animal experiments were performed in accordance with the guidelines approved by the Meeting of the Institutional Animals Ethics Committee, held at the Jawaharlal Nehru University (JNU), approval code no. 19/2014, and also at the International Centre for Genetic Engineering and Biotechnology (ICGEB), New Delhi, India (approval no. ICGEB/IAEC/08/2016/TACF-JNU). We also followed guidelines issued by the Department of Biotechnology, Government of India. All mice used for experiments were ethically euthanized by asphyxiation in carbon dioxide according to institutional and Department of Biotechnology regulations.

### Mice.

Six- to eight-week-old female C57BL/6 mice were purchased from the animal house facility of the ICGEB. All mice used for experiments were housed under barrier conditions in the Tuberculosis Aerosol Challenge Facility (TACF) of the ICGEB and treated humanely according to speciﬁed animal care protocols of TACF and ICGEB guidelines.

### Preparation of curcumin nanoparticles.

Nanocurcumin was prepared according to methods described in our previous report (24). Briefly, 10 g of curcumin (Sigma, USA) was dissolved in 2.5 liters of distilled ethanol at room temperature and filtered to obtain a clear solution. This solution was then stirred in a high-speed homogenizer (T 25 digital Ultra-Turrax) at 12,000 to 15,000 rpm, and the required volume of Milli Q water containing 0.1% citric acid (Merck, India) was added to it slowly over a period of 1 h until the ethanol concentration reached 40% (vol/vol) and curcumin particles started to precipitate from the solution. The entire suspension was then homogenized over ice in a high-pressure homogenizer (Avestin C5 high-pressure homogenizer) at 30,000 lb/in^2^ for 30 cycles. The aqueous suspension was then made to 0.1% polysorbate 80 (Sigma, USA), homogenized at 12,000 to 15,000 rpm (T 25 digital Ultra-Turrax; IKA, USA) again for 1 h, and filtered. The filtered slurry was dried at 80°C in an oven to obtain curcumin powder. The particle size was determined by using a high-resolution transmission electron microscope (JEM 2100F; JEOL, USA).

### Bacterial culture.

All mycobacterial strains were grown in 7H9 medium (Middlebrook; Difco) supplemented with 10% albumin, dextrose, and catalase enrichment medium (OADC; Difco, USA) with 0.05% Tween 80 and 0.2% glycerol, and cultures were grown to mid-log phase. Aliquots of the cultures in 20% glycerol were preserved at −80°C, and these cryopreserved stocks were used for infections.

### BCG immunization experiments.

Mice were immunized subcutaneously with 1 × 10^6^ CFU of BCG in 100 μl of sterile phosphate-buffered saline (PBS). After 1 day of rest, these mice were treated with curcumin nanoparticles at 10 mg/kg of body weight every day in 100 μl of PBS, administered intraperitoneally (i.p.) for a total of 30 days. Mice were subsequently rested for 30 days. They were then challenged via the aerosol route with M. tuberculosis strain H37Rv, as described below, and organs were harvested after euthanasia to determine the bacterial burden and for immunophenotyping at different time points. For lymphocyte proliferation assays, mice were sacrificed 75 days after aerosol challenge. Seventy-two hours prior to sacrifice, animals were injected intraperitonially with BrdU (Sigma) dissolved in 100 μl of PBS.

### M. tuberculosis low-dose aerosol infection of mice.

M. tuberculosis strain H37Rv (ATCC 27294; American Type Culture Collection) was a kind gift from Colorado State University. Mouse infections were performed in accordance with the low-dose aerosol infection model, using a Madison aerosol chamber (University of Wisconsin, Madison) with the nebulizer precalibrated at approximately 90 to 120 CFU per mouse. For aerosol infection, cultures were washed twice with PBS and made into a single-cell suspension by passage through a 26-gauge needle 10 times. Next, 15 ml of the M. tuberculosis H37Rv single-cell suspension (100 × 10^6^ cells) was placed into the nebulizer reservoir of the Madison aerosol chamber, calibrated to deliver the desired CFU of bacteria into the lungs of mice kept in the chamber in 15-min cycles. Twenty-four hours after aerosol challenge, 3 mice were euthanized for quantitation of pathogen delivery to lungs by measurement of CFU in lung homogenates. Mice were found to be infected with approximately 110 CFU of M. tuberculosis in their lungs. The mice were maintained under biosafety level 3 containment thereafter.

### Quantiﬁcation of pathogen burden by CFU counts.

Randomly selected mice were euthanized at various time points, after which organs were harvested and homogenized in 0.2-μm-ﬁltered PBS. Lungs and spleen cell homogenates were plated on 7H11 plates containing 10% OADC and incubated at 37°C for approximately 21 days. CFU were counted, and the pathogen burdens in lungs and spleen were estimated.

### *In vitro* experiments with peritoneal macrophages.

Six- to eight-week-old C57BL/6 mice were given an i.p. injection of 1 ml thioglycolate medium (4%). After 5 days, macrophages were obtained by peritoneal lavage. Macrophages were washed once with cold PBS and suspended in cold RPMI 1640 medium. Cells were counted, seeded on 12-well plates, and maintained at 37°C in RPMI 1640 medium supplemented with penicillin-streptomycin (1,000 U/ml) and 10% heat-inactivated fetal calf serum. After overnight incubation, nonadherent cells were washed off. Adherent cells were infected with H37Rv at a ratio of 10:1. Cells were then treated with 60 nM curcumin nanoparticles and kept at 37°C in a CO_2_ incubator. After 24 and 48 h of infection, macrophages were harvested for CFU determination and flow cytometry.

### Flow cytometry.

**(i) Surface and intracellular staining of peritoneal macrophages.** After 48 h of infection, peritoneal macrophages were harvested for flow cytometry. Four to six hours prior to staining, phorbol myristate acetate (PMA) (50 ng/ml) and ionomycin (750 ng/ml) were added to the cultures. Moreover, brefeldin A was also added to these cultures at this time point for intracellular staining, as described below.

**(ii) Surface and intracellular staining of *in vivo* samples.** Spleens and lungs were isolated from mice and macerated by using frosted slides in ice-cold RPMI 1640 medium (Gibco, Invitrogen) containing 10% fetal bovine serum to prepare a single-cell suspension. Red blood cells were lysed with lysis buffer by incubation at room temperature for 1 to 2 min and then washed with RPMI 1640 medium. The cells were counted, and 1 × 10^6^ cells per well were cultured for staining in 12-well plates. Lymphocytes were stimulated with H37Rv complete soluble antigen (CSA) and cultured overnight before staining. For staining of antigen-presenting cells, no such CSA challenge was performed. Before staining, cells were washed twice with fluorescence-activated cell sorter (FACS) buffer and then stained with antibodies directed against surface markers. After the addition of antibodies, cells were kept for 40 min at room temperature for optimum staining. Next, cells were washed again with buffer and resuspended in FACS buffer. For intracellular staining, lymphocytes (2 × 10^6^ cells) were cultured in 12-well plates and stimulated with H37Rv complete soluble antigen overnight, with 5 μg/ml of brefeldin A (BioLegend) being added during the last 6 h of culture. For intracellular staining, cells were ﬁxed after surface staining with 100 μl of ﬁxation buffer (BioLegend) for 30 min and then washed twice with permeabilization buffer (BioLegend), resuspended in 200 μl of permeabilization buffer (BioLegend), and stained with ﬂuorescently labeled antibodies. After staining, cells were washed again with FACS buffer and resuspended in FACS buffer. The ﬂuorescence intensity of ﬂuorochrome-labeled cells was measured by ﬂow cytometry (FACS-LSR Fortessa; BD Biosciences). BD FACSDiva software was used to acquire the cells, and data analysis was performed using FlowJo software (TreeStar).

### Histological studies.

Pieces of lungs from mice 75 days after infection were perfused, removed, fixed in 4.5% formalin for 12 h, and transferred to 70% ethanol; paraffin blocks were then made; and 6-μm sections were cut and stained with hematoxylin and eosin according to our previous work (58).

### Statistical analysis.

All data were derived from ≥3 independent experiments. Statistical analyses were conducted using GraphPad software, and values are presented as means with standard deviations (SD). Signiﬁcant differences between the group means were determined by analysis of variance (ANOVA) with Tukey’s *post hoc* analysis. Differences were considered statistically signiﬁcant at a *P* value of <0.05.

## Supplementary Material

Supplemental file 1
