# Supplementary material for: Curcumin Nanoparticles Enhance Mycobacterium bovis BCG Vaccine Efficacy by Modulating Host Immune Responses
Source: Infect Immun. 2019 Oct 18;87(11):e00291-19. doi: 10.1128/IAI.00291-19 (PMC6803339; doi:10.1128/IAI.00291-19)
Supplement: Supplemental file 1 [file IAI.00291-19-s0001.pdf]

Supplementary Table 1: Nano-curcumin increases the TCM: TEM ratio both in Spleen and lungs of BCG Immunised mice.

		CD44-CD62L gated population	H37Rv	NC+H37Rv	BCG+H37Rv	BCG+NC+ H37Rv
Spleen	$CD4^+$	T_{CM}/T_{EM}	0.39	0.24	0.45	0.57
	$CD8^+$	T_{CM}/T_{EM}	0.3	0.27	0.37	0.52
Lungs	$CD4^+$	T_{CM}/T_{EM}	0.1	0.10	0.16	0.24
	$CD8^+$	T_{CM}/T_{EM}	0.14	0.15	0.2	0.26

		CD44-CCR7 gated population	H37Rv	NC+H37Rv	BCG+H37Rv	BCG+NC+ H37Rv
Spleen	$CD4^+$	T_{CM}/T_{EM}	0.36	0.38	0.46	0.78
	$CD8^+$	T_{CM}/T_{EM}	0.24	0.25	0.34	0.56
Lungs	$CD4^+$	T_{CM}/T_{EM}	0.32	0.4	0.37	0.53
	$CD8^+$	T_{CM}/T_{EM}	0.35	0.47	0.64	0.73

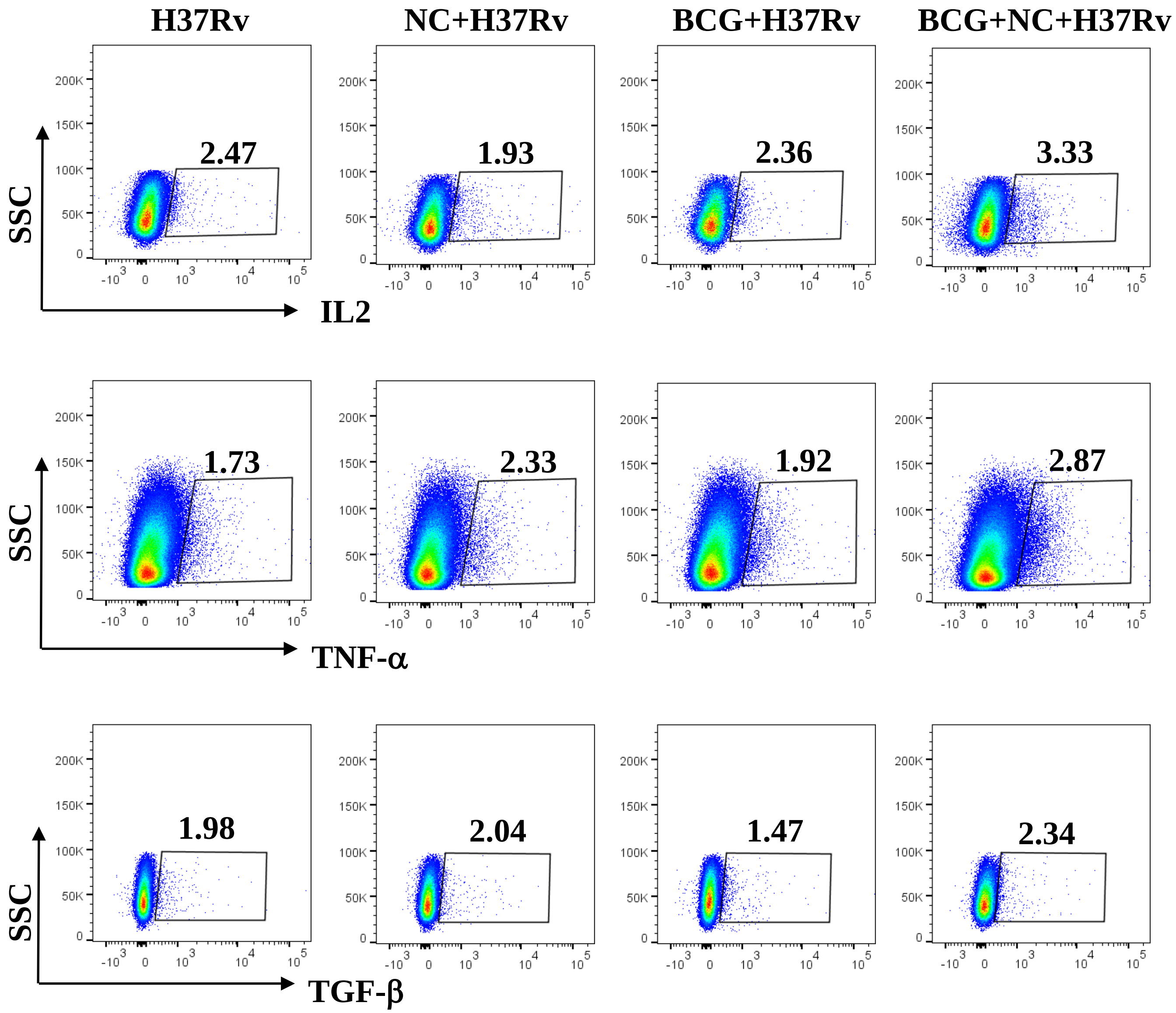

Supplementary Fig. 1 : Representative pseudo colour **plots of IL-2- , TNF-α- and TGF-β-cytokine producing cells in splenocytes of different experimental groups**.
